# Impact of Digital Phenotypes and Question-Asking on Emotional Disorders in Adolescents: 4-Week Field Study

**DOI:** 10.2196/66536

**Published:** 2025-10-07

**Authors:** Minseo Cho, Doeun Park, Myounglee Choo, Doug Hyun Han, Jinwoo Kim

**Affiliations:** 1 Human Computer Interaction Lab, School of Business, Yonsei University, 50, Yonsei-ro, Seodaemun-gu, Building 212Seoul, 03722, Republic of Korea; 2 School of Management of Technology, Human Computer Interaction Lab, Yonsei University, Seoul, Republic of Korea; 3 HAII Corporation, Seoul, Republic of Korea; 4 Department of Psychiatry, College of Medicine, Chung-Ang University, Seoul, Republic of Korea

**Keywords:** adolescent, emotional disorder, digital phenotype, digital health, field study, EMA, self-monitoring, ecological momentary assessment

## Abstract

**Background:**

Adolescence is the period with the highest incidence of mental disorders, with approximately one-third, half, and two-thirds of cases emerging by ages 14, 18, and 25 years, respectively. Proactive interventions are essential, and digital phenotyping has emerged as a promising approach for timely detection and management. However, passive digital phenotyping is limited to sensor-detectable behaviors, while active phenotyping is often confined to clinical scales, missing the opportunity to capture users’ subjective perspectives and emotional nuances. Furthermore, the potential therapeutic effect of the data collection process itself on emotional disorder management remains underexplored.

**Objective:**

This study developed and tested a mobile app that collects passive and active digital phenotypes related to adolescents’ emotions and daily behaviors. The study aimed to assess the app’s impact on managing emotional disorders through self-monitoring and to identify daily lifestyle indicators that can predict and track the development of such disorders.

**Methods:**

A 4-week parallel, nonequivalent control group design was employed. The intervention group installed a digital phenotype collection tool on their mobile devices for 28 days. Passive data (location, sleep, and screen time) were continuously recorded. Active data were collected through ecological momentary assessments delivered randomly up to 8 times daily, prompting participants to report their current mood and levels of depression, anxiety, and stress. The control group received no intervention. Both groups were assessed at time points on emotional disorders, self-efficacy, and time management. Postintervention interviews were conducted with the intervention group.

**Results:**

Thirty-six Korean adolescents participated (19 control, 17 intervention). The intervention group showed significant reductions in depression (*P*=.04, *d*=0.42) and stress (*P*=.03, *d*=0.46) and improvements in self-efficacy (*P*=.002, *d*=0.50) and time management abilities (*P*<.001, *d*=0.39), with small to large effect sizes. No significant change was observed in anxiety levels (*P*=.11). Correlational analysis revealed weak but significant links between passive digital phenotypes and daily emotional states.

**Conclusions:**

Integrating active and passive digital phenotypes through a mobile collection tool can help manage emotional disorders in adolescents. Use of the tool was associated with moderate reductions in depression and stress, as well as improvements in self-efficacy and time management, while anxiety levels remained unchanged, possibly due to adolescents’ differing perceptions of anxiety. Passive digital phenotypes such as location variability and phone usage showed modest correlations with daily emotional states, supporting their potential as ecological markers. These findings suggest that digital phenotype collection not only aids in monitoring but may also have therapeutic benefits by promoting self-reflection on mood and behavior. High adherence rates further support the practicality and acceptability of this approach for long-term emotional disorder management in adolescents.

## Introduction

### Background

Alarming statistics reveal that nearly half of all mental illnesses begin before the age of 18 years, placing adolescents at the highest risk of developing emotional disorders [1,2]. Depression and anxiety are the most diagnosed conditions in adolescents, affecting nearly every aspect of their lives. These conditions can disrupt school, strain family relationships [3], and fuel risky behaviors such as smoking, drinking, and substance abuse. Most disturbingly, depression is a leading risk factor for suicide among adolescents [4]. Adolescence is a critical period of physiological, psychological, and social development; as such, mental health during this phase is intricately linked to well-being in adulthood [5]. This underscores the urgent need for early diagnosis and timely intervention in adolescent emotional disorders.

Digital phenotypes, the real-time collection of physiological and behavioral data through computer-based tools, have gained attention as a means to prevent and address mental disorders [4]. By capturing continuous, comprehensive data often unavailable through traditional methods, digital phenotypes offer new opportunities for the early detection and treatment of emotional disorders. These phenotypes can be categorized as active and passive. Active digital phenotypes require conscious user input, such as responses to self-reported surveys. Passive digital phenotypes collect data unobtrusively through smartphone sensors like accelerometers and Global Positioning System (GPS) as individuals go about their daily lives [6]. Research shows that digital phenotypes can effectively detect the severity of social anxiety, predict depression recurrence [6], identify suicidal ideation, and even prevent symptom deterioration with real-time monitoring [7].

However, opinions vary on whether passive digital phenotypes are sufficient to understand and explain emotional disorders. Context is crucial in shaping an individual’s emotional perceptions and reactions, yet passive digital phenotypes often fail to capture this nuanced information. While behaviors such as movement or physical activity can be easily inferred from GPS and accelerometer data, more complex activities or emotional states that require inferences or are inaccessible to sensors remain unknown through passive sensing alone.

To gain a deeper understanding of emotional disorders, active digital phenotypes must be used to complement passive methods [8]. However, existing research primarily relies on clinical scales that may not fully account for the contextual factors relevant to emotional disorders [6,9-13]. Moreover, much of the literature treats digital phenotypes merely as data collection outcomes [14,15]. In contrast, active digital phenotyping functions as a form of self-monitoring, empowering individuals to gain insights into their emotional responses and develop targeted strategies to improve their mental health [16]. Therefore, the process of collecting active digital phenotypes may itself contribute to addressing emotional disorders.

In this context, our study is among the first to integrate both passive and active data collection in a single tool and to deploy it in the real world among adolescents. This dual-mode approach enables the collection of context-rich, ecologically valid data that neither passive nor active methods alone can provide. By analyzing the relationship between these digital phenotypes and daily assessed emotional disorder levels, we aim to determine how passive and active digital phenotypes function as complementary, parallel indicators of adolescent emotional health. Furthermore, this study investigates how the process of data collection can promote emotional disorder levels, self-efficacy, and time management skills.

### Related Works

#### Digital Phenotypes and Emotional Disorders

Passive digital phenotypes are data collected about people’s daily lives using digital devices [6]. This definition contains 4 further elements [9]. First, it involves continuous data collection and analysis rather than collecting data at specific time points. Second, the individual is the unit of analysis. Third, data are collected in natural, uncontrolled environments to capture real-world behavior. This point distinguishes passive from active digital phenotypes, such as ecological momentary assessments (EMAs), which will be discussed later. Fourth, passive digital phenotypes can be collected by devices that individuals already own, such as smartphones, without additional instruments [9].

A variety of physiological and behavioral passive digital phenotypes can be used to identify abnormal behavioral patterns associated with mental disorders [6,10]. People with mental disorders exhibit symptoms that affect mobility, life satisfaction, and social interactions, some of which can be quantified through digital phenotypes [11,12]. Symptoms of depression include negative affect, hopelessness, devaluation of life, and inertia, while anxiety is characterized by physiological arousal such as sweating, trembling, increased heartbeat, and avoidance tendencies [13]. Tracking these symptoms with passive digital phenotypes can allow for the early diagnosis and timely treatment of certain diseases, thereby more effectively managing health.

Adolescence is a particularly vulnerable developmental stage, often characterized by exposure to various stressors and their debilitating mental health effects [17]. Stress is accompanied by physical, psychological, or social complaints or dysfunctions resulting from individuals feeling unable to meet requirements or expectations placed on them. Stress during adolescence is associated with negative outcomes such as decreased well-being, impaired mental health, anxiety, and depression [17-20]. These challenges often co-occur, and symptoms of one disorder may predict the concurrent or future development of other mental health disorders. Therefore, addressing stress and emotional disorders is crucial for promoting long-term mental health.

Digital phenotyping could be particularly effective in addressing adolescent mental health challenges as adolescents often lack self-awareness or face communication barriers, particularly due to externalizing psychiatric disorders [21]. Given that approximately 95% of adolescents own or have access to smartphones, most by age 11 years, these technologies could be especially effective at tracking and intervening in mental health issues [22]. Mobile sensing and digital phenotypes can support health behaviors by enhancing individuals’ responsibility for their health and have been shown to increase health literacy [23]. These technologies also promote stronger relationships between health care providers and adolescents by facilitating meaningful conversations based on collected data, enabling timely treatment and encouraging help-seeking behaviors [24].

#### Ecological Momentary Assessment

An EMA [25] is used to build a comprehensive understanding of individuals [26,27]. It collects real-time self-reports of individuals’ behaviors, thoughts, and emotions in their daily lives [28]. EMAs examine the present moment and so are conducted repeatedly over an extended period. With advancements in technology, EMAs are often implemented through digital platforms, such as smartphones and wearable devices [25].

Emotions are not fixed or stable; they are highly responsive to immediate circumstances and daily experiences [29]. Traditional data collection methods that rely on retrospective reports may struggle to capture these dynamics due to several biases, such as recency and novelty effects, which may compromise their validity [30]. EMAs overcome this limitation by gathering data at the moment when these experiences occur. This real-time approach allows for a detailed examination of how emotions evolve throughout the day [31].

To effectively manage emotional disorders, it is necessary to understand the conditions that trigger them [32]. Clinical scales and interviews with health care providers rarely address the context of the symptoms. In contrast, EMAs solicit a wide range of information from individuals, such as their location, time, and interpersonal influences, which can identify specific contexts in which emotional disorders are triggered [25,33]. Such detailed information can be used to better manage these conditions.

Unlike passive digital phenotypes, which rely on data collected by sensors, EMAs can directly ask individuals about their subjective experiences, which allows them to capture contextual data that sensors may not be able to [33]. Active and passive digital phenotypes complement each other, and neither is inherently superior. By integrating EMAs with passive digital phenotypes, researchers can achieve a more comprehensive understanding of emotional disorders [34]. This study collected active digital phenotypes through EMAs and passive digital phenotypes to develop detailed insights into the contextual factors influencing emotional disorders in adolescents.

#### Self-Monitoring and Self-Efficacy

Self-monitoring is the process in which individuals observe and record their own behaviors, emotions, and thoughts and adjust themselves accordingly [35]. Through this process of data collection and reflection, individuals can enhance their self-awareness and choose to engage in behaviors to achieve specific goals. Emotional self-monitoring helps identify factors that influence mood, both positively and negatively, allowing individuals to develop strategies to improve their mental health [36]. Self-efficacy is an individual’s belief in their ability to plan and execute actions to achieve desired outcomes [37] and is closely linked to mental health. By boosting self-efficacy, self-monitoring can enhance mental health, increasing an individual’s belief that they can autonomously manage and overcome emotional challenges [38,39].

Previous studies have identified several benefits of self-monitoring. Regularly measuring symptoms of emotional disorders can help predict their recurrence and evaluate the effectiveness of the ongoing treatments [40]. It provides opportunities to reflect on the context surrounding symptom occurrences, aiding in understanding the causes. Moreover, self-monitoring helps identify factors that either alleviate or worsen emotional disorders, providing insights into their patterns and impacts [41]. Finally, self-monitoring is done independently without external interventions, so it encourages individuals to actively participate in managing their mental health [36,42,43], which can enhance an individual’s agency, positively influencing their self-efficacy.

Combined with these benefits, self-monitoring has been shown to improve mental health symptoms [44]. Studies on adolescents in the early stages of depression have demonstrated that self-monitoring gives them insights into their health, which is the first step in a stepped-care approach [33,45,46]. Furthermore, self-monitoring extends symptom-free periods in patients with depressive disorders [47] and helps them focus on behaviors that can help them control their emotional disorders [48]. Even for individuals not clinically diagnosed with emotional disorders, self-monitoring has been shown to have positive effects, such as motivating them to pursue health management [49].

To effectively define the role of digital phenotypes in managing emotional disorder, it is crucial to understand them as self-monitoring. However, the role of digital phenotypes as a tool for self-monitoring has not been investigated yet. This study explored whether digital phenotypes could help individuals engage in self-monitoring of emotional disorders. Accordingly, a service was developed for this study that integrates the collection of digital phenotypes, which have been validated for their use in the early diagnosis and treatment of adolescent mood disorders. The study evaluated the effects of this service on adolescents’ depression, anxiety, stress, and self-efficacy levels and time management abilities.

## Methods

### Study Design

Participants were not randomly assigned due to constraints related to the study tool’s operational environment as the app was only compatible with Android smartphones. Given the limited number of adolescents using Android devices who were available to participate, it was not feasible to recruit a sufficiently large sample for both groups exclusively from Android users. The study used a 4-week parallel, nonequivalent control group design.

The intervention group consisted of Android users who installed and used the app for 28 days. The control group, which was not required to use the app, had no restrictions on smartphone type. Both groups were assessed on key variables at baseline (day 0), midpoint (day 14), and postintervention (day 28). Following the intervention, semistructured interviews were conducted with the intervention group to explore their experiences using the digital phenotype collection tool.

Interviews were conducted via the Zoom online conferencing tool within 1 week after the intervention period ended. With participants’ consent, all interviews were audio-recorded, transcribed verbatim, and then deleted to maintain confidentiality. Topics covered the perceived impact of app usage on personal life and mood and suggestions for improvement. Data were analyzed using thematic analysis, with 2 researchers independently coding to identify recurring themes and patterns.

### Recruitment

This study included individuals who: (1) were South Korean residents in the age group of 12‐18 years, (2) were enrolled in middle or high school, (3) spoke Korean as their native language and had no difficulty communicating in it, and (4) understood the study purpose and voluntarily agreed to participate. Participants were recruited to the intervention group if they owned an Android smartphone and had no issues using it to ensure the efficient operation of research tools.

Participants were recruited through multiple channels, including digital flyers posted on social media platforms (eg, Instagram, KakaoTalk channels), online youth community forums, and outreach via collaborating clinicians. Recruitment materials briefly explained the study purpose and provided a link to an online preassessment survey. Those who completed the survey received the download link to the study tool via a text message.

This recruitment strategy may have introduced selection bias as participants were more likely to be adolescents who actively engage with digital platforms and possess higher digital literacy. Additionally, the requirement for Android smartphone ownership in the intervention group may have introduced systematic differences between groups, particularly in terms of socioeconomic status and smartphone usage patterns.

### Sample Size Calculation

The sample size was calculated using a 2-sided significance level of .05, a statistical power of 80%, 2 groups, and 3 repeated measures. Based on these parameters, a sample size of 26 participants per group was estimated to be sufficient to detect an effect size of .33 in the reduction of depression levels. This effect size was selected based on findings from a systematic review of digital mental health interventions targeting young people, which reported small to moderate effect sizes for similar outcomes. To account for a potential 5% dropout rate, we increased the target sample size to 56 participants in total.

### Intervention

The study tool was an app that collects digital phenotypes validated as predictive indicators for emotional disorders. The app was developed based on exploratory research into adolescents’ intentions and behaviors in voluntarily recording emotions and daily activities [50]. To develop the tool, we first observed how adolescents recorded target active digital phenotypes regarding emotions and lifestyle behaviors within a digital environment and inquired about the tools they used, including their advantages and disadvantages.

These observations were then translated into system design goals, which included designing interfaces that resemble digital planners, streamlining data input processes with shortcut interactions, providing visual reports on collected data to reward users’ efforts, and writing friendly EMA prompts. By aligning the system’s features with users’ natural behaviors, the app was designed to enhance the quality of collected active digital phenotypes.

**Figure 1.**
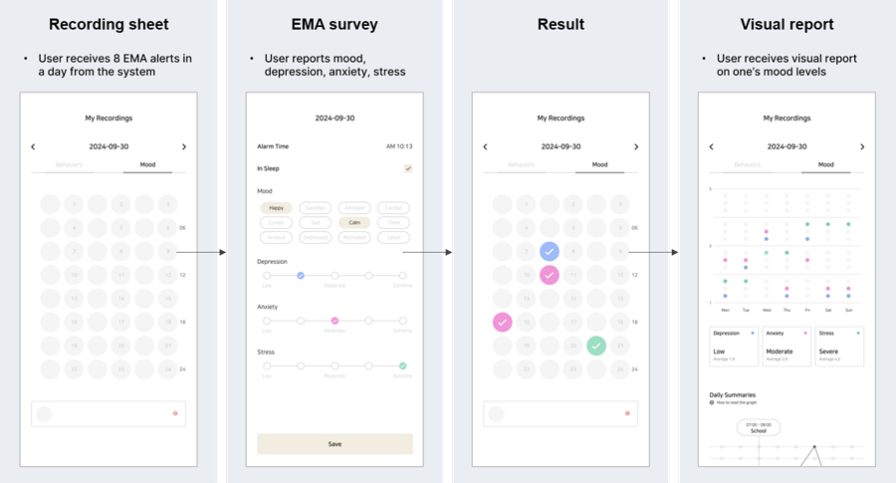
Process of mood recording with corresponding system interface screens. EMA: ecological momentary assessment.

Emotional recording followed an EMA methodology where users responded to notifications delivered randomly throughout the day (Figure 1). These notifications were sent upon waking, before school, during lunchtime, after school, before private tutoring, after private tutoring, before bedtime, and if the phone was used for more than 30 minutes after midnight. Users were restricted to recording their emotions only when prompted by these notifications.

**Figure 2.**
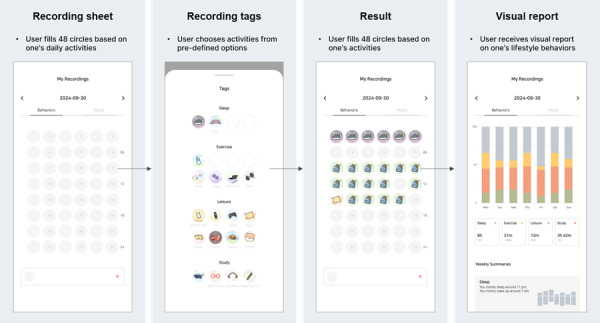
Process of lifestyle behavior recording with corresponding system interface screens.

The recording of lifestyle behaviors was facilitated through a screen displaying a table that divided the 24-hour day into 30-minute intervals, resulting in 48 cells (Figure 2). By clicking on each cell, users could access a pop-up screen with sleep, exercise, leisure, and study categories, each of which included specific activities, like napping or meeting friends. The recorded data were then processed into reports, allowing users to gain detailed insights into the structure of their daily lives and their levels of emotional disorders.

### Measures

#### Demographic Information

Participants provided demographic information, namely gender, age, level of education, and address. The level of effort typically invested in mood and life management was assessed using a 5-point Likert scale, ranging from 1 (“very low”) to 5 (“very high”).

#### Korean Version of the Depression Anxiety Stress Scale

The Korean version of the Depression Anxiety Stress Scale, adapted by Lee (2002) from the version shortened by Henry of the original developed by Lovibond, was used to measure levels of depression, anxiety, and stress [51-53]. This shortened scale consists of 21 items, with 7 items assessing each symptom. Each item is rated on a 4-point Likert scale from 0 (“does not apply at all”) to 3 (“applies most of the time”), with higher scores indicating greater severity of symptoms.

#### Self-Efficacy Scale

Self-efficacy was measured using a scale developed by Sherer and translated by Kim [54,55]. The scale includes 22 items divided into 3 subfactors: confidence, self-regulatory efficacy, and preference for task difficulty. Each item is rated on a 5-point Likert scale from 1 (“strongly disagree”) to 5 (“strongly agree”), with higher scores indicating higher self-efficacy. The internal consistency coefficient, Cronbach α, for this scale is 0.88.

#### Time Management Ability Scale

Time management ability was evaluated with a scale developed by Jeong and Jang, which includes 30 items categorized into 3 subfactors: planning ability, execution ability, and evaluation ability [56]. Participants rated each item on a 5-point Likert scale from 1 (“not at all”) to 5 (“always”), with higher scores indicating better management ability. The internal consistency coefficients, Cronbach α, for these subfactors are 0.91, 0.86, and 0.89, respectively.

#### mHealth App Usability Questionnaire

To evaluate the usability of the app developed for this study, we used a self-reported scale that assesses the usefulness and usability of mobile health apps [57]. The scale consists of 21 items rated on a 7-point scale from 1 (“strongly disagree”) to 7 (“strongly agree”). The questionnaire is divided into 3 subfactors: ease of use and satisfaction, assessed by 8 items, system information arrangement, assessed by 6 items, and usefulness, assessed by 7 items.

#### Passive Digital Phenotypes

The location digital phenotypes, collected using the devices’ GPS functions, included total distance traveled, total travel time, location variance, entropy, and normalized entropy. These data were preprocessed by estimating travel speed through time derivatives. Using a threshold of 1 km/h, the data were classified as either stationary or moving [57,58]. The stationary data were analyzed with the K-means clustering algorithm to identify the locations where participants spent most of their time.

The sleep digital phenotypes included total amount of sleep, total daytime sleep, and total nighttime sleep. Considering the daily routine and schedule of adolescents, daytime was defined as 7 AM to 11 PM and nighttime as from 11 PM to 7 AM. Sleep states were estimated using data collected from cameras, microphones, accelerometers, and smartphone log data, considering factors such as ambient light, noise, and smartphone screen time [59].

Digital phenotypes calculated from screentime data contained in device logs included total phone usage duration, total daytime phone usage, total nighttime phone usage, total phone lock duration, and the frequency of phone unlocks [60]. We handled outliers using the interquartile range method, where values outside 1.5 times the IQR were considered outliers and excluded. We did not attempt to impute missing values as the data were passive and imputation could introduce artifacts.

#### Active Digital Phenotypes

Mood records were collected using EMAs. System notifications were delivered randomly throughout the day, except during school, private tutoring, and sleep. Upon receiving a notification, participants recorded their current mood by selecting the mood-related adjectives (eg, happy, sad, relaxed) and rated their levels of depression, anxiety, and stress on a scale from 1 (“very low”) to 5 (“very high”).

Participants recorded their daily activities using predefined options. Key lifestyle areas include sleep, exercise, leisure, and study-related activities. Each 24-hour day was divided into 30-minute intervals and participants were asked to select the corresponding activity for each time slot (eg, studying, walking). The recorded lifestyle behaviors were organized by type, start time, and end time.

### Statistical Analysis

Analyses were conducted using SPSS 26.0. Descriptive statistics were used to describe and compare participants’ baseline characteristics. Univariate analyses were performed to compare baseline values using the *t* test for independent samples. A 2-way ANOVA model followed by Scheffé post hoc tests was used to test the effect of app usage on each variable. Data were analyzed using 2×3 repeated measures ANOVAs with 1 between-subject factor (intervention group and control group) and 1 within-subject factor (preintervention, midintervention, and postintervention). The statistical significance level was defined as *α*=.05.

### Ethical Considerations

This study was reviewed and approved by the Yonsei University Institutional Review Board (IRB no. 7001988-202406-HR-2045-05). All participants provided written informed consent after receiving a full explanation of the study purpose and procedures from the research team. Participant confidentiality was maintained throughout the study, with each individual assigned a numerical code according to their order of participation. Upon completion of the study, participants received compensation of 100,000 KRW (US $72.00).

## Results

### Descriptive Statistics

Of the 21 participants in the intervention group, 17 completed the study, resulting in an 81% retention rate. Dropouts occurred due to technical issues (n=2), failure to complete the preintervention survey (n=1), and personal reasons (n=1). In the control group, all 19 participants completed the study, resulting in a 100% retention rate. The intervention group consisted of 3 male and 14 female participants, with a mean age of 16.70 (SD 0.98) years. The control group included 2 male and 17 female participants, with a mean age of 16.63 (SD 1.02) years. There were no statistically significant differences in gender distribution or age between the two groups.

At baseline, there were no statistically significant differences between the groups in anxiety, self-efficacy, or time management abilities. However, the intervention group showed significantly higher levels of depression and stress. For depression, the control group had a mean score of 10.21 (SD 2.20), while the intervention group had a mean score of 18.12 (SD 3.60); this difference was statistically significant (*t*_34_=–2.25, *P*=.03). For stress, the control group’s mean score was 12.00 (SD 3.10) and the intervention group’s score was 19.76 (SD 3.80). A Mann–Whitney *U* test indicated a significant difference between the groups (*U*=234.5, *P*=.02).

**Figure 3.**
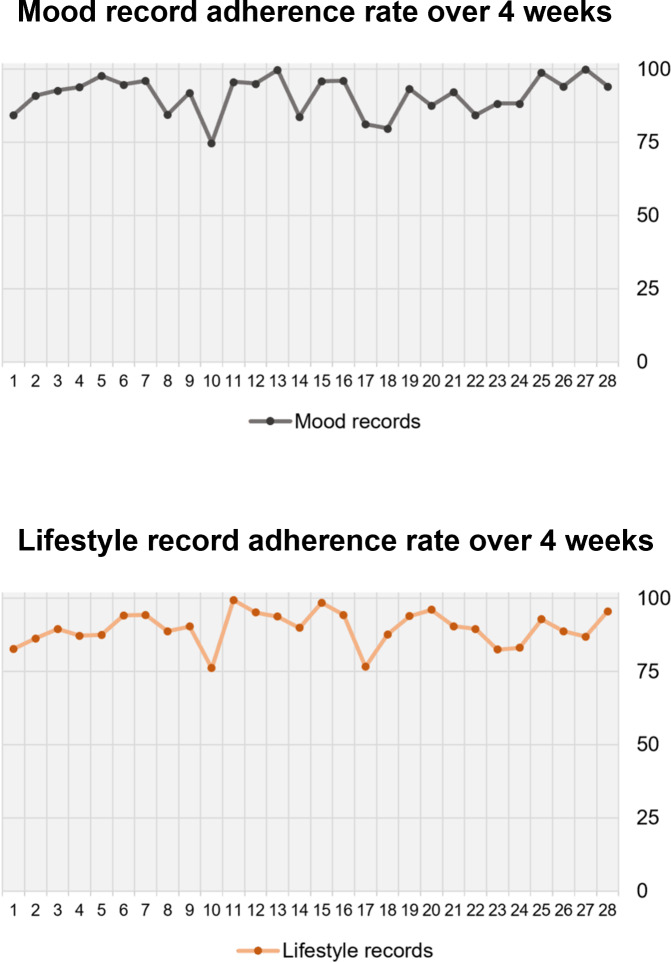
Average adherence rates for mood and lifestyle recording over 4 weeks.

Adherence to lifestyle records was defined as the percentage of records made from the 48 requests. Adherence to mood records was defined as the percentage of responses to the 28 daily EMA alerts sent. Lifestyle record adherence had an average of 89.68% and a range of 71.5%‐99.6% (Multimedia Appendix 1). Mood record adherence had an average of 90.65% and a range of 79.3%‐99.6%. The average adherence rates per participant indicate that most participants complied with the system’s requests for collecting digital phenotypes (Figure 3).

Intervention group satisfaction was assessed using the mHealth App Usability Questionnaire at the end of the intervention. The survey consisted of 21 items rated on a 7-point scale, evaluating the tool’s ease of use, user satisfaction, system information arrangement, and usefulness. Participants’ responses showed a high level of satisfaction, with an average score of 6.31 of 7. The median score was 6.29, indicating that half of the participants rated the tool at or above this value.

Scores ranged from a minimum of 5.81 to a maximum of 6.86, demonstrating relatively low variability in user evaluations. The SD was 0.31, further confirming consistent positive feedback across the participant group. These findings suggest that the digital phenotype collection tool was generally well-received by adolescents, both in terms of usability and usefulness, which is also reflected in the high adherence rate throughout the intervention period [57].

### Changes in Repeated Measures

**Figure 4.**
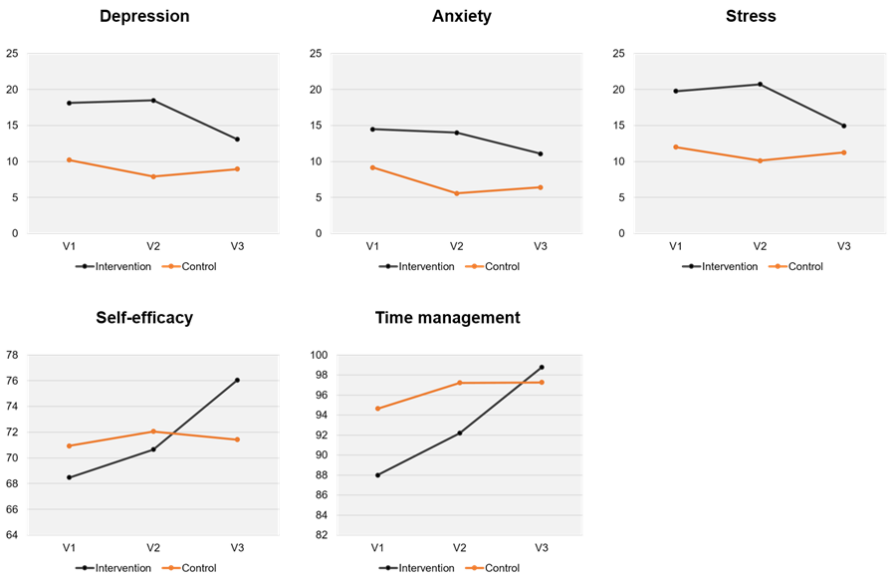
Repeated measures ANOVA results comparing the intervention and control groups.

A repeated measures ANOVA revealed a significant main effect of time (*F*_2, 68_=3.62, *P*=.04), indicating that depression levels changed significantly across the 3 time points, regardless of group. A repeated measures ANOVA revealed a statistically significant interaction effect between time and group for depression levels (*F*_2, 68_=3.65, *P*=.04), indicating that the intervention group experienced a different trajectory of change compared with the control group (Figure 4). For anxiety, although a significant main effect of time was observed (*F*_2, 68_=5.64, *P*=.009), the interaction effect was not significant (*F*_2, 68_=2.35, *P*=.11), suggesting similar changes across both groups.

In contrast, stress levels showed no significant main effect of time (*F*_2, 68_=2.56, *P*=.07), but there was a significant interaction between time and group (*F*_2, 68_=3.55, *P*=.03). Time management ability significantly improved over time (*F*_2, 68_=11.54, *P*<.001), with a significant interaction effect (*F*_2, 68_=4.78, *P*=.01), indicating a greater improvement in the intervention group. Similarly, self-efficacy scores showed both a significant main effect of time (*F*_2, 68_=6.99, *P*=.002) and a significant interaction effect (*F*_2, 68_=5.27, *P*=.007). These results suggest that the intervention had a positive and differential effect on depression, stress, time management, and self-efficacy when compared with the control group.

### Passive Digital Phenotypes and EMA

We analyzed the relationship between passive digital phenotypes and emotional disorder levels over time. While previous studies often clustered subjects based on their baseline emotional disorder scores, this study used ecologically assessed emotional disorder levels collected over a 4-week period [61,62]. This approach aimed at the same timeframe of the data in the analysis and to capture the dynamic nature of an individual’s emotional disorders, which fluctuate within a day.

Collected passive digital phenotypes were categorized by participants and averaged daily, resulting in 28 sets of passive digital phenotypes per participant. Outliers and missing values were excluded from the analysis. The results of the analysis are presented in Tables1^3^, summarizing Pearson correlation coefficients with a significance level of α=.05.

**Table 1.** The correlation analysis of passive digital phenotypes and depression ecological momentary assessment.

	Depression	*P* value
Location		
Total travel distance	−0.0932^a^	.04
Total travel time	−0.0806	.08
Location variance	0.1041^a^	.02
Location entropy	−0.1186^b^	.01
Normalized location entropy	−0.1089^a^	.02
Sleep		
Total sleep duration	−0.0053	.91
Daytime sleep duration	0.0988^a^	.03
Nighttime sleep duration	−0.0429	.35
Phone log		
Total phone usage time	−0.1123^a^	.01
Total phone screen lock time	0.0980^a^	.03
Daytime phone usage time	−0.0511	.27
Nighttime phone usage time	−0.1421^b^	.002
Phone screens unlock frequency	−0.1511^c^	<.001

a *P*<.05.

b *P*<.01.

c *P*<.001.

**Table 2.** The correlation analysis of passive digital phenotypes and anxiety ecological momentary assessment.

	Anxiety	*P* value
Location		
Total travel distance	−0.0782	.09
Total travel time	−0.0735	.11
Location variance	0.1376^b^	.003
Location entropy	−0.1234^b^	.007
Normalized location entropy	−0.1296^b^	.005
Sleep		
Total sleep duration	0.0452	.33
Daytime sleep duration	0.1342^b^	.003
Nighttime sleep duration	−0.0057	.90
Phone log		
Total phone usage time	−0.9078^c^	<.001
Total phone screen lock time	−0.1159^a^	.01
Daytime phone usage time	−0.0401	.38
Nighttime phone usage time	−0.1329^b^	.004
Phone screens unlock frequency	−0.1159^a^	.01

a *P*<.05.

b *P*<.01.

c *P*<.001.

**Table 3.** The correlation analysis of passive digital phenotypes and stress ecological momentary assessment.

	Stress	*P* value
Location		
Total travel distance	−0.0156	.73
Total travel time	0.0118	.80
Location variance	0.0955^a^	.04
Location entropy	−0.0566	.22
Normalized location entropy	−0.0896	.05
Sleep		
Total sleep duration	0.0281	.54
Daytime sleep duration	−0.1262^b^	.006
Nighttime sleep duration	−0.0197	.67
Phone log		
Total phone usage time	−0.0881	.06
Total phone screen lock time	0.0694	.13
Daytime phone usage time	−0.0461	.32
Nighttime phone usage time	−0.1074^a^	.02
Phone screens unlock frequency	−0.1798^c^	<.001

a *P*<.05.

b *P*<.01.

c *P*<.001.

For location digital phenotypes, there were no statistically significant correlations between total travel time and emotional disorders. Total travel distance was negatively correlated with depression (*r*=−0.0932, *P*=.04). Statistically significant positive correlations emerged between location variance and depression (*r*=0.1041, *P*=.02), anxiety (*r*=0.1376, *P*=.003), and stress (*r*=0.0955, *P*=.04). Location entropy was negatively correlated with depression (*r*=−0.1186, *P*=.01) and anxiety (*r*=−0.1234, *P*=.007), while normalized location entropy was statistically significantly negatively correlated with depression (*r*=−0.1186, *P*=.01) and anxiety (*r*=−0.1089, *P*=.02).

Regarding sleep digital phenotypes, there were no statistically significant correlations between total sleep duration, nighttime total sleep duration, and the level of emotional disorders. However, daytime total sleep duration showed statistically significant positive correlations with depression (*r*=0.0988, *P*=.03), anxiety (*r*=0.1342, *P*=.003), and stress (*r*=0.1262, *P*=.006). These results suggest that as daily levels of emotional disorders increase, daytime sleep duration or nap duration also tends to increase.

The relationships between digital phenotypes of phone use and daily depression, anxiety, and stress levels are presented in Tables1^3^. Statistically significant correlations were found between all variables except for daytime phone usage. Total phone usage exhibited a statistically significant negative correlation with depression (*r*=−0.1123, *P*=.01) and anxiety levels (*r*=−0.9078, *P*<.001). Phone screens unlock frequency showed statistically significant negative correlations with depression (*r*=−0.1151, *P*<.001), anxiety (*r*=−0.1159, *P*=.01), and stress levels (*r*=−0.1798, *P*<.001).

Total phone screen lock time showed a statistically significant positive correlation with depression (*r*=0.0980, *P*=.03) and negative correlation with anxiety (*r*=−0.1159, *P*=.01), whereas nighttime phone usage time exhibited statistically significant negative correlations with depression (*r*=−0.1421, *P*=.002), anxiety (*r*=−0.1329, *P*=.004), and stress levels (*r*=−0.1074, *P*=.02). These findings suggest that as daily levels of emotional disorders increase, nighttime phone usage decreases. Overall phone usage tends to decrease on days with higher depression and anxiety levels, while total phone screen lock time increases on days with higher anxiety levels.

## Discussion

### Main Results

#### Overview

This study developed and deployed a tool that captures both passive and active digital phenotypes in real-world settings to build a comprehensive dataset for understanding emotional disorders in adolescents. The tool collects behavioral data commonly used in prior studies of passive digital phenotypes, such as location, sleep patterns, and phone usage, and extends data collection into domains that are not easily captured by sensors, including study and rest routines, self-reported mood, and subjective emotional distress.

Unlike studies that focused solely on passive sensing or retrospective self-reports, this study positioned self-monitoring as both a data collection strategy and an intervention mechanism. As participants recorded their emotions, they became more aware of their levels of depression, anxiety, and stress and examined the underlying causes of these feelings. This process caused participants to integrate their reflections on both emotions and daily life, allowing them to construct causal relationships between daily experiences and emotional states based on the gathered digital phenotypes. This aligns with prior findings that self-monitoring can facilitate emotional awareness and behavior change, but this study extends that work by integrating real-time active digital phenotypes with passively collected context.

Furthermore, the correlation analysis between passive and active digital phenotypes revealed patterns consistent with earlier findings. For instance, higher late-night phone use and irregular sleep patterns were associated with elevated stress and depression levels. These observations suggest that integrated digital phenotype systems can surface meaningful, co-occurring patterns between lifestyle behaviors and emotional states among adolescents. The strength of this study is further supported by the high adherence rate across both active and passive data streams. Overall, the findings demonstrate how combining passive sensing with active self-reporting can generate a comprehensive understanding of emotional well-being and support light-touch interventions through self-reflection.

#### User-Centric Design for Digital Phenotypes

As demonstrated in this study, having individuals consistently record their mood and lifestyle behaviors can aid in managing emotional disorders by leveraging the benefits of self-monitoring. While developing our study tool, we applied exploratory research to enhance both the quantity and quality of active phenotype data. A pilot study with adolescents helped us understand how their daily routines and digital habits could inform a design aligned with their lived experiences.

Based on these insights, we implemented a timetable-based interface that reflected the structure of a typical adolescent day, allowing participants to intuitively locate and record emotions in the context of their daily schedules. We designed context-aware EMA nudges that prompted emotion recordings at relevant points in the day, increasing compliance without causing notification fatigue. The visualization of the collected data prioritized the types of insights adolescents reported to value most, making the reflection more meaningful.

The impact of these design features is reflected in the tool’s high usability and strong adherence. Our findings suggest that in designing tools to collect active digital phenotypes from adolescents, it is essential to find an appropriate balance between the burden of self-reporting and the perceived value of data reflection. A well-designed interface that minimizes friction and maximizes personal relevance can significantly improve both engagement and the quality of self-reported data.

#### Digital Phenotypes and Personalized EMAs

The potential of digital phenotype collection as an intervention also emerged using EMA notifications. In follow-up interviews, participants reported that when EMA alerts were tailored to their daily life context rather than generic reminders, they experienced a stronger sense of social support and were more motivated to engage with the tool. The effect was particularly notable when prompts included words of encouragement and comfort, such as cheering them on to attend school or private tutoring sessions. Additionally, the tool issued EMAs during extended periods of late-night device usage. Participants interpreted these messages as expressions of care, which encouraged them to reflect on their behavior and, in many cases, reduce nighttime screen time.

In this study, EMA notifications were primarily time-based and followed a fixed template designed by the researchers. However, by integrating both passive and active digital phenotypes, EMA prompts could be further personalized to match each user’s real-time context. For example, if an adolescent logs study time that exceeds recommended limits, the tool could deliver a supportive message acknowledging their effort while gently encouraging balance. Likewise, if an increase in physical activity is detected, a message might inquire about their experience, reinforcing awareness and positive habits. Such personalization could be significantly enhanced by generative artificial intelligence, which would allow EMA content to evolve dynamically based on ongoing user interactions and behavioral patterns.

This personalized, context-aware approach can extend to facilitating more proactive interventions. By analyzing trends in digital phenotype data when heightened distress is reported, we can infer individual patterns related to emotional disorders. For instance, this study found a positive correlation between increased nap duration and elevated emotional disorder levels. If the system detects more frequent or longer naps, it could adjust EMA notification such as, “You’ve been napping more than usual; have you been feeling more down lately?” However, it is important that these prompts are framed with care. Messages should invite introspection rather than make assumptions and always be mindful of user privacy, especially when dealing with sensitive behavioral data.

Our findings extend the current discourse on digital phenotypes in 2 significant ways. First, EMA can be more than simply assessments by indirectly helping to alleviate emotional disorders. EMAs involve frequent interactions between the system and the user, with each session shaping the user experience. In this study, we particularly designed empathetic survey prompts, which helped build rapport with and provide emotional support to participants. Second, this study showed how passive digital phenotypes, which are commonly used to determine the timing of EMA prompts, could be used. Participants viewed the tool’s apparent interest in them as a major benefit, which suggests that passive digital phenotypes could be used more broadly to optimize timing and personalize other aspects of EMAs based on users’ behavioral and psychological characteristics.

### Limitations

This study has several limitations. First, although it included both an intervention group that used a tool for 4 weeks and a control group that did not use it, participants were not randomly assigned to the groups and there was a gap in recruitment timing. The tool was exclusively designed for Android phones, which limited our ability to randomly assign participants to groups. While we attempted to ensure homogeneity by considering factors such as age, gender, residential area, and grade, there may have been unexamined characteristics that could have influenced the study results.

Second, active digital phenotypes were collected via self-reporting, which was only possible when participants were not otherwise engaged. To accurately assess participants’ daily routines, the study needed to be conducted during school terms but not around major school events, such as national examinations, which could affect levels of depression, anxiety, and stress. Thus, there was a 1-month difference in the start times between the intervention and control groups, which might have affected the results. Third, there were statistically significant differences in depression and stress level baseline scores between the two groups. The intervention group reported higher levels of both depression and stress initially. This discrepancy indicates that the intervention group had greater potential for improvement, so the positive impact of the digital phenotype collection service should be interpreted with caution.

Fourth, there was an uneven distribution of gender and grade among participants. Female participants were more represented than male participants in both groups, and there were more high school students than middle school students. These imbalances may limit the generalizability of the findings to male and middle school students. However, emotional disorders are generally more frequent and severe among female adolescents, so this distribution may reflect real-life conditions. Additionally, as the study was conducted exclusively with Korean adolescents, cultural factors unique to Korea may have influenced participants’ behaviors and responses. This cultural specificity could limit the applicability of the findings to adolescents in other cultural contexts. Future studies should aim for a more balanced representation by gender and grade and include more diverse cultural settings to improve the generalizability of the results.

Fifth, this study did not specifically target adolescents diagnosed with emotional disorders. Although some participants had high emotional disorder scores, these scores did not necessarily equate to clinical diagnoses. Furthermore, the EMAs assessed depression, anxiety, and stress levels using single items for ease of response, which may not fully represent the participants’ clinical status. Future research should focus on participants with clinically diagnosed emotional disorders to enhance the generalizability and clinical relevance of the study findings.

### Conclusions

This study explored how digital phenotypes can be used to manage emotional disorders among adolescents. To this end, we developed a digital phenotype collection tool that integrates active digital phenotypes into EMAs for mood disorders and related daily contexts. This study included a 4-week field test to investigate the potential diagnostic and therapeutic roles of active digital phenotypes to assess how participating in digital phenotype collection affects the emotional disorders of depression, anxiety, and stress; self-efficacy; and time management abilities. We also examined the relationships between passive digital phenotypes and adolescents’ daily emotional states.

The results showed that tool use was negatively correlated with depression and stress levels and positively correlated with self-efficacy and time management abilities. However, it was not correlated with anxiety levels and interviews with participants provided insights as to why. Adolescents understood depression in a way that matched clinical definitions, such as lethargy and sadness. However, adolescents tend to interpret anxiety in a more situational manner, associating it with specific stressors like upcoming examinations or incomplete homework, rather than recognizing it as a clinical condition. This discrepancy might explain why no significant improvement in anxiety levels was observed when assessed using clinical scales.

Furthermore, we analyzed the relationship between passive digital phenotypes and their relationships with adolescents’ levels of depression, anxiety, and stress via EMAs. This correlation analysis revealed that location variance, location entropy, daytime sleep duration, phone usage time, and phone screens unlock frequency were weak but statistically significantly correlated with daily levels of depression, anxiety, and stress, which is like trends observed in other studies [4,63]. Unlike past research that typically compared baseline scores, our study used long-term, ecologically assessed data, showing that EMAs can be used to establish predictive correlations with passive digital phenotypes.

These findings suggest that digital phenotype collection services can be used to understand and manage adolescent emotional disorders. Despite the recognized potential of digital phenotypes for this purpose, few studies have collected active digital phenotypes in the context of emotional disorders, and none have focused on their effectiveness in managing these disorders. This study provides preliminary evidence that the use of a digital phenotype collection tool, particularly through the process of reflecting on both mood and lifestyle-related behaviors, may contribute to improvements in depression, self-efficacy, and time management among adolescents. These findings suggest that the integration of passive and active digital phenotypes could play a supportive role in better understanding and potentially addressing emotional challenges in this population. Additionally, the high level of adherence maintained throughout the study, despite the extensive user participation required, indicates that the methods used to collect digital phenotypes were successful.

## Supplementary material

10.2196/66536 Multimedia Appendix 1 Average adherence rates for mood and lifestyle recording per participant.

## Abbreviations

EMA: ecological momentary assessment
