# Supplementary figures and images for: Impact of Digital Phenotypes and Question-Asking on Emotional Disorders in Adolescents: 4-Week Field Study
Source: JMIR Hum Factors. 2025 Oct 7;12:e66536. doi: 10.2196/66536 (PMC12505407; doi:10.2196/66536)

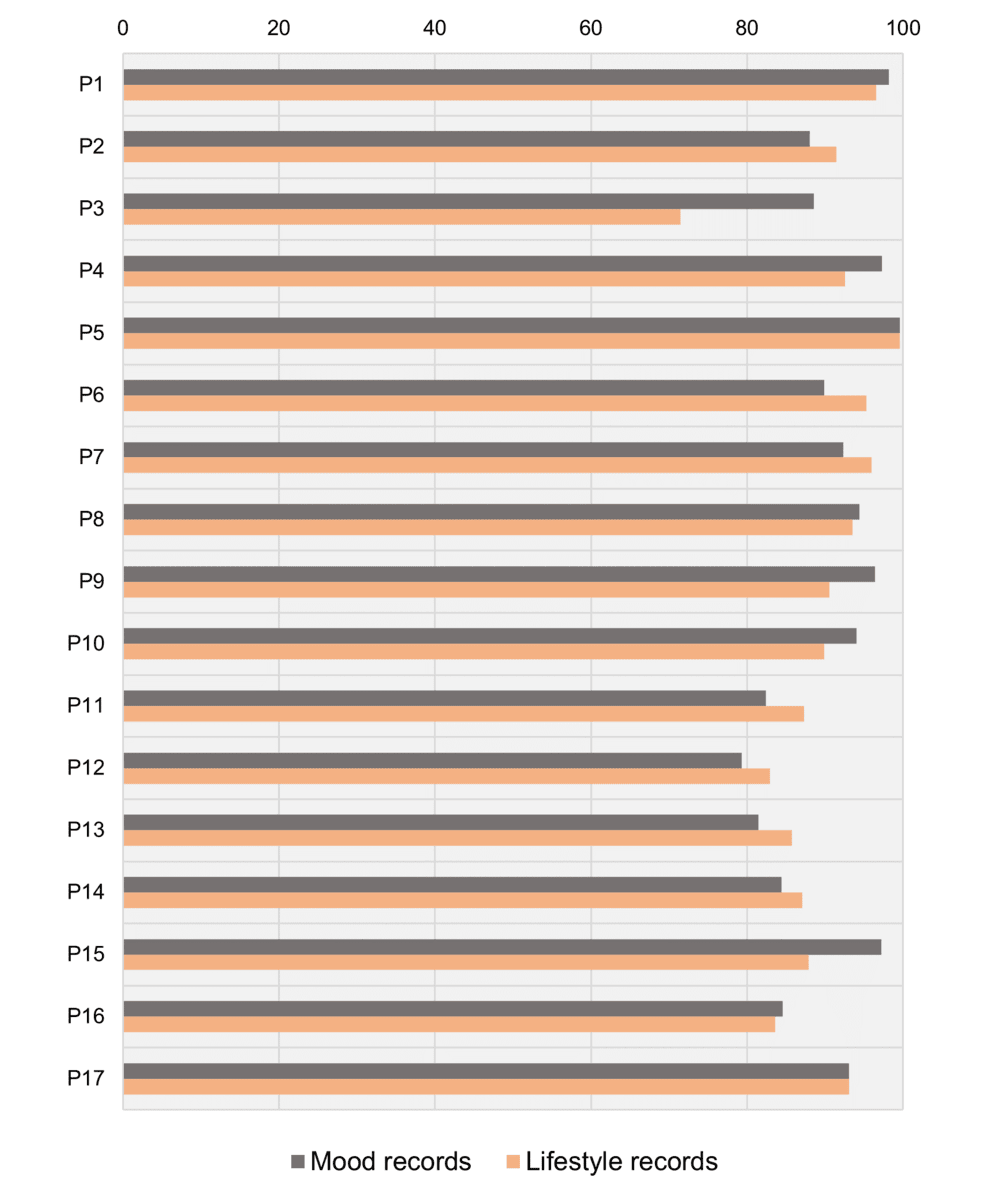

Supplement: Multimedia Appendix 1 [file humanfactors-v12-e66536-s001.png]
